# Insights on Place and Psychological Dynamics for Private Forest Owners’ Management Transitions: A Study on Increasing the Share of Broadleaves in Sweden

**DOI:** 10.1007/s00267-025-02234-x

**Published:** 2025-08-08

**Authors:** Louise Eriksson, Caroline Rapp

**Affiliations:** Department of Geography, Umeå University, Umeå, Sweden

**Keywords:** Behavioral change, *Betula Pendula*, Silver birch, Native fast-growing broadleaves, Place dimensions

## Abstract

Due to the increased risk of forest damage associated with climate change, forest management needs to be diversified. To encourage a transition towards a diversified forest, facilitating and hindering factors need to be understood. This study uses a novel approach to study how place dynamics and psychological factors promoting a change are associated with change intentions among private forest owners—a group with a significant role in management transitions. The goal intention to change tree species composition and three behavioral intentions to increase the share of broadleaves were examined using a survey of a randomly selected sample of private forest owners in Sweden (*N* = 1793). Results revealed that intentions to increase broadleaves through natural regeneration were stronger than the intention to adopt improved silver birch, supporting a higher potential in changes more aligned with current practices. Physical, social, and relational dimensions of place were separately and interactively associated with change intentions. For example, owners in the south region, with a greater information flow and a higher trust in forest actors, displayed a stronger intention to adopt improved birch, and trust was more important among non-certified compared to certified owners. Damage risk perceptions and psychological drivers (e.g., goal feasibility) were associated with change intentions. In addition, results confirmed the role of experience, cognitions, and emotions associated with improved birch for the intention to adopt the species. This interdisciplinary approach draws attention to the dynamics in the owners’ decision context and decision-making to understand the unfolding of forest management transitions.

## Introduction

With increased risk of damage to forests due to a changing climate and current management, there is a need to diversify the forest to increase biodiversity and resilience, thereby making the forest less vulnerable to damage (Jandl et al. 2019; Lindner et al. 2014). The forest management can be diversified through more close-to-nature forestry including natural regeneration and continuous cover forestry (CCF), or through more intensive forestry, including practices such as short rotations and artificial regeneration with improved reproductive material (Roitsch et al. 2023). In conifer dominated forests, an increase in broadleaves is favorable for biodiversity and may be appreciated for recreation (Agimass et al. 2018; Dubois et al. 2020; Grönlund et al. 2020; Hornigold et al. 2016). In addition, fast-growing broadleaves are used for energy, pulp, and wood products (Dubois et al. 2020; Ibarra et al. 2021; Rytter and Lutter 2021). Since native fast-growing broadleaved species can facilitate biodiversity (Grönlund et al. 2020), using improved seed material of such species to ensure better quality trees for production (including faster growth) may ensure diverse ecosystem services and is thus one viable option for increasing the share of broadleaves.

The management of forests is influenced by ecological factors such as climate and soil conditions, but also the social system, including regulations, policy, and the market, with forest owners, forest companies, and the government as pivotal actors (Blanco et al. 2017). In several European countries (e.g., Sweden and Finland) and in the United States of America, a large share of the forest land is owned by non-industrial private forest owners (also labelled family forest owners) (Luke 2019; National Association of State Foresters 2024; Swedish Forest Agency (SFA) 2024). Major changes in management are thus dependent on the decisions made by this group of owners. To facilitate a transition towards a diversified forest, there is a need to understand how feasible different management options are and factors encouraging or preventing such changes (Eriksson and Sandström 2022; Hertog et al. 2022; Husa and Kosenius 2021). Whereas the determinants of forest owners’ management have been examined (Eriksson and Fries 2020; Floress et al. 2019), the understanding of factors that are promoting or hindering changes in management is inadequate. In addition, the place and time dynamics associated with management decision-making has generally not been considered. This is a limitation since the owners make their decisions in a physical location but also as part of a social setting where certain policies are in place (e.g., certification), with local (e.g., neighboring owners) and regional (e.g., ownership organizations) connections, as well as following interactions with forest actors across scales. Contemplating a change in management is furthermore likely to be a stepwise process. This study addresses this gap by examining these dynamics among private forest owners in Sweden in relation to the potential for increasing the share of broadleaves. Such an approach may guide the development of policy and decision support systems by illustrating how the same policy or intervention may operate differently depending on place characteristics.

### Factors Important for Forest Management Among Private Forest Owners

Different climate and soil conditions offer diverse possibilities for changing forest management, and management varies across regions. For example, owners in the south of Sweden (in the hemiboreal and nemoral zones) have been found to display a higher management activity for production and climate adaptation compared to owners in the north (in the boreal zone) (Blennow 2012; Eriksson and Fries 2020). Other place related factors such as whether the owner is living on the estate or not, have shown small or mixed impacts on management (Coté et al. 2016; Eriksson and Fries 2020; Silver et al. 2015). Studies have also revealed that the owners’ social network is associated with harvest activities (Sagor and Becker 2014). Moreover, certification, in interaction with membership in a forest owner association, has implications for management, with those being both a member and certified showing the highest management activity (Bergkvist et al. 2024; Westin et al. 2023). Psychological factors such as knowledge, forest values and risk perceptions, attitudes, norms, and beliefs linked to management approaches, are confirmed determinants of management decisions (Eriksson and Fries 2020; Fischer and Denny 2024; Floress et al. 2019; Sousa-Silva et al. 2018). Although socio-demographic factors have been found to be less important for management decisions than psychological factors (Ficko et al., (2019); Vulturius et al. 2018), owners of larger forest estates commonly display higher levels of management, including harvesting and planting (Coté et al. 2016; Floress et al. 2019). Women often show a lower management activity than men (Coté et al. 2016; Eriksson 2018c; Lidestav and Berg Lejon 2013), but other socio-demographic variables, such as age, tend do display small or mixed effects on management activities (Eriksson and Fries 2020; Husa and Kosenius 2021).

Actual management behaviors, stated behaviors, and intentions have been examined to learn about forest management behaviors. While management intentions do not reflect actual management (Silver et al. 2015), studies of intentions are important to learn about the potential for adopting new management approaches. By acknowledging that behavioral change is a process and recognizing when and why intentions are likely to translate into actual behavior (Bamberg 2013), studies of intentions are valuable for understanding transitions. Previous studies have examined intentions to use diverse alternative management strategies (Fischer 2019) and covered determinants of owners’ general intention to engage in climate-adapted forestry (Vulturius et al. 2018). However, there is scarce knowledge of what determines intentions to use different types of strategies in the future.

### Theoretical Framework

**Fig. 1:**
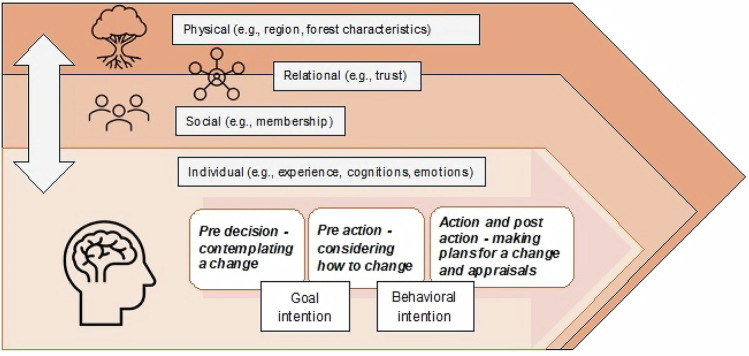
Physical, social and relational place dynamics and the decision-making process preceding change in forest management

The terms transition and transformation have been used interchangeably to depict a change (often more radical) from one state to another (Hölscher et al. 2018). However, transition is frequently used to refer to the process of change and transformation to a change of greater magnitude (Child and Breyer 2017). Change in one sub-system (e.g., energy) is often examined in transition research, while transformation research commonly covering change in both the natural and social systems (Hölscher et al. 2018). This study examines the potential for a transition in forest management towards increased diversity (i.e., the change process in one subsystem), yet dependent on, and with implications for, both ecological and social systems. The focus is on understanding change at the individual level yet dynamically connected to diverse dimensions and scales through the concept of progressive sense of place proposed by Massey (1991), and using the initial part of the stage model of self-regulated behavioral change developed by Bamberg (2013) to outline psychological processes preceding change (Fig. 1).

A progressive sense of place (Massey 1991) depicts that there is a multiplicity of perspectives within one place, that places are continuously developed, and defined both by the place itself as well as other places (Barron et al. 2020). To integrate a progressive sense of place with sustainability transitions, Sonnino and Milbourne (2022) outline an integrated view of the natural and social dimensions of place, the fluidity of place, social relations within places (e.g., power struggles), and interactions with other places as key dimensions. Linkages and connectivity between different dimensions of place are explicitly emphasized, suggesting that different place dimensions and scales cannot be considered in isolation when attempting to understand a transition. According to this view, the physical characteristics (e.g., country, region) make up only one dimension of the forest owners’ decision-making context. Social factors, including networks the owner belongs to and operating policy mechanisms (e.g., market and informational policies), as well as relationships with other owners and forest actors (e.g., the government) are additional layers making up the place in which owners make decisions. For example, a member of an owner organization is situated in a different social context than non-members, even though they may have neighboring forests, but even two owners that are both members may interact differently with other forest actors, with implications for how much they each trust others. The physical, social, and relational dimensions of place are also evident at different scales, from local to regional and national. The importance of progressive views of place have been emphasized in other studies of people—environment interactions (Raymond et al. 2018; West et al. 2020).

The process leading to a decision to change management can be captured by psychological factors. The stage model of self-regulated behavioral change (Bamberg 2013) portrays individual behavioral change as a transition through different stages involving goal setting, making plans to achieve the goal, and appraisal processes. In the first phase, the decision to change has not been made yet but the individual is contemplating whether a change is desired and feasible. The individual may subsequently form a goal intention (e.g., an intention to change tree species) and move to the second phase. During this phase, different ways of achieving the goal are considered and may result in a decision to choose one option over others reflecting the formation of a behavioral intention (e.g., intention to plant improved birch). The subsequent phases include planning for the change, forming an implementation intention regarding where, how and when to perform the change and subsequently trying and evaluating the new behavior. At any time, the individual may return to a prior phase. Early in this process the individual must acknowledge the need for a change, but change is also facilitated by favorable normative influences (e.g., what would be an acceptable change) and high perceived feasibility of achieving the goal for example. Perceptions of others’ behaviors signaling what is accepted behaviors, labelled social descriptive norm, and perceptions of what is the right thing to do reflecting a personal norm are two types of normative influences (Thøgersen 2006). The formation of behavioral intentions is also dependent on how the specific behavioral option is experienced, interpreted, and evaluated, including different cognitions (thoughts and beliefs) and emotions (Eagly and Chaiken 1993). Overall, we propose that place dimensions are important for psychological processes of change. Place constitutes the setting where change is elaborated on and the extent to which, as well as how, different place variables encourage change thereby determine the outcome of this process, in combination with the individual’s response.

### The Present Study

The aim of this study is to examine how place dynamics and psychological factors promoting change are associated with change intentions among private forest owners in Sweden. In addition to natural regeneration of broadleaves, improved reproductive material for native species may be a viable option to ensure the delivery of diverse ecosystem services, including biodiversity and wood products. Adopting the native silver birch (*Betula Pendula*) with improved qualities achieved through breeding (hereafter improved birch) is one such example. We examine the goal intention to change tree species composition and three behavioral intentions including two changes aligning with current management, increased broadleaved and mixed forest through natural regeneration, and one novel change represented by the adoption of improved birch (Objective 1). Departing from a progressive sense of place, we also examine how place dimensions covering physical (region), social (certification, membership in forest owner association), and relational (trust in forest actors, flow of information) dimensions are separately, and interactively important for change intentions (Objective 2). The decision to change is also believed to be directed from within, initiated by the realization that a change is needed. Hence, we examine the role of damage risk perceptions, but also factors encouraging a change (social and personal norms) and enabling a change (goal feasibility) (i.e., general psychological variables) for the goal intention and the behavioral intentions. In addition, we examine the role of variables reflecting the owners’ perspective on improved birch, covering experience, subjective knowledge, attitudes, emotions, and negative beliefs (i.e., specific psychological variables) for intention to adopt improved birch (Objective 3).

## Methods

### Study Area

Sweden is 69% covered by forests. Productive forests make up 84% of the forest land and are dominated by conifers, primarily Norway Spruce (*Picea abies*) and Scots Pine (*Pinus sylvestris*). Broadleaved forest and mixtures of conifers and broadleaves make up 8% and 7.5% of the productive forest, respectively. Birch is the most common broadleaved species comprising 17.5% of the timber volume, and is mainly naturally regenerated (Rytter 2019; Official Statistics of Sweden 2023). Native to forests in northern Europe, it is appreciated due to its importance for biodiversity (Felton et al. 2021), and while birch is mainly used for pulp in Sweden, it can be used for e.g., sawn wood, veneer, and non-wood products such as birch sap, but potentially to a greater extent also timber in the future (Dubios et al. 2020). The availability of improved reproductive material for one of the dominant birch species, silver birch, is low but increasing.

Improved silver birch needs to be planted and is not in widespread use; it can therefore be considered a novel management option in this context. In 2023, just below 310,000 individual private forest owners owned 48% of the productive forests (SFA 2024). Since 1993, the production and environmental goals have been considered equally important in the Swedish forest policy (Gov. Bill 2007/08:108) and in the latest government bill the need to increase forest growth and secure biomass was highlighted along the necessity of protecting and developing biodiversity in forests (Gov. Bill 2021/2022:58). Freedom among forest owners to make management decisions remained high and ownership rights were strengthened in this bill.

### Study Design and Respondents

A survey directed at a randomly selected sample of individual private forest owners (owning 5 ha or more of forest land) in Sweden was conducted. To avoid increasing problems with non-reach respondents (and a potentially reduced response rate), the age limit was set to 20–80 years. However, due to a mistake in the initial sample selection, the sample frame was older than requested, and an additional sample was therefore added. In the end of 2023, 5000 owners received the questionnaire also including owners over the age of 80, with an additional 1000 owners only in the age range 20–80 years receiving the questionnaire in the beginning of 2024 (net sample = 5951). Data was collected using a commercial survey company (PFM Research). Ethical guidelines as stipulated in the 1964 Declaration of Helsinki were followed during data collection. The owners were informed about the purpose of the study and how personal information was handled. They were also told that participation was voluntary before consenting to participate. To protect the privacy of participants, data were pseudonymized before analyses. Since no sensitive personal information was handled as defined in Swedish legislation (the Ethics Review Act 2003:460), no ethical approval was needed.

The total response rate was 30.3% (*n* = 1793) following in total five contacts (invitation by post to answer digitally, paper questionnaire, sms reminder, and two reminders by post including paper questionnaire). The majority answered the paper questionnaire (77.4%). Mean age was 74 years (25–102 years), and 28% were women. In total, 39% had a university education. The owners were almost evenly distributed between Norrland in the boreal zone (19% in the north and 15% in the south), Svealand in the hemiboreal zone (29%) and Götaland partly in the hemiboreal zone and partly in the nemoral zone (36%). The majority of the owners, 71%, lived in a rural area with less than 10,000 residents, but less than half, 40%, lived on their forest property. The average forest holding was 71.4 hectares (SD = 154.9) (5–20 ha: 33%, 21–50 ha: 30%, 51–200 ha:31%, >200: 6%) and the average size of agricultural land was 8 hectares (SD = 22.2). Half of the owners stated that they were members in a forest owner association, and just below one third (30%) that they were certified according to FSC, PEFC or both. The sample of owners was older than the population (mean age in population 2023: 61 years), a larger share was men (share of women in population: 38%) and fewer were owners with small forest estates (5–20 ha: 44%, 21–50 ha: 29%, 51–200 ha: 23%, >200: 4%) (SFA 2024). To control for these deviations, data were weighted on gender, age, and size of forest land in the analyses (descriptives for the weighted sample are displayed in the Supplementary material, Table S1). The impacts of these deviations are further considered in the “Discussion” section.

### Measures

The questionnaire was developed through a co-creation process. Forest researchers and stakeholders from seven organizations (e.g., owner and network organizations) taking part in the competence center “Trees for me” focusing on fast-growing broadleaves in Sweden contributed with diverse perspectives. The process was led by the authors, and the forest researchers and stakeholders were given the opportunity to comment on what questions to include. Previous research on forest management was also utilized to guide the development of questions. In the questionnaire, improved birch was described as having better qualities (e.g., straighter stems) and faster rotation (15–20%, around 5–10 years shorter) compared to the naturally generated silver birch, and that its use in Sweden is so far limited but much more common in the neighboring country of Finland. Information on gender, age, size of forest land, size of agriculture land, and region where the forest property is located was taken from the owner register. In addition, questions on whether the owner live in a rural or urban place and on their property or not were included in the questionnaire. Concepts and measures used to assess place dimensions and psychological factors, as well as change intentions, are described in Table 1, including references supporting the operationalization of concepts and descriptive data.

**Table 1:** Concepts, measures and descriptives

Concepts	Measures
Place: physical	
Region	From register
Place: social	
Membership	What of the following correspond to your forest? Member in a forest owner association (e.g., Norra, Södra, Mellanskog)
Certification	What of the following correspond to your forest? Certification according to Forest Stewardship Council (FSC), Certification according to Programme for the Endorsement of Forest Certification (PEFC)
Place: relational	
Trust (4, *α* = 0.68) *M* = 3.20, SD = 0.80	What level of trust do you experience in relation to the following when it comes to advice about forest management? Private forest owners, Forest companies, The Swedish Forest Agency, Forest researchers Five-point scale (1 = No trust at all, Great trust) (Eriksson 2018a)
Flow of information (8, *α* = 0.78) *M* = 1.82, SD = 0.68	How often do you retrieve information from, or are inspired by the following in the management of your forest? Others in the household or co-owners, Other forest owners, Home page, newspapers or reports from forest owner associations, Homepage, newspapers (e.g., Skogseko, or reports from The Swedish Forest Agency, Advisors/timber byers at forest owner associations, Advisors at the Swedish Forest Agency, Advisors at other organizations such as private consulting businesses, Others' forest stands (through seeing/visiting) Five-point scale (Every week, Several times a month, Several times a year, Yearly, More seldom or never). Reversed scale. (André et al. 2017)
General psychological factors	
Damage risk perception (3, *α* = 0.69) *M* = 2.83, SD = 0.88	How likely do you perceive it to be that your forest would be impaired by the following in a time period of 10 years? Extensive damage by storm, snow, frost or fire (so called abiotic damages), Extensive damage by insects (e.g., the spruce bark beetle) or fungi (e.g., root rot, rust), extensive damage by wild game (e.g., moose). Five-point scale (1 = Not at all likely, 5 = Very likely) (Eriksson 2018a)
Social norms broadleaved species (single item) *M* = 2.04, SD = 0.92	Other forest owners close to me increase the share of broadleaves in their stands. Five-point scale (1 = Completely disagree, 5 = Completely agree) (Thøgersen 2006)
Personal norms broadleaved species (2, α = 0.87) *M* = 2.19, SD = 1.11	I perceive a personal responsibility to increase the share of broadleaves. I would have a bad conscious if I did not increase the share of broadleaves. Five-point scale (1 = Completely disagree, 5 = Completely agree) (Steg et al. 2011)
Goal feasibility broadleaved species (2, *α* = 0.79) *M* = 3.11, SD = 1.02	I think it would be easy to increase the share of broadleaves in my forest. To me it is completely possible to increase the share of broadleaves. Five-point scale (1 = Completely disagree, 5 = Completely agree) (Bamberg 2013)
Specific psychological factors: Improved birch	
Experience (single item) *M* = 1.65, SD = 0.93	How often do you see stands dominated by the following? Improved birch Five-point scale (1 = Never, 3 = Sometimes, 5 = Very often).
Subjective knowledge (single item) *M* = 1.56, SD = 0.90	How much knowledge do you believe that you have about the management of the following broadleaves? Five-point scale (1 = No knowledge at all, 2 = Little knowledge, 3 = Some knowledge, 4 = Quite a lot of knowledge, 5 = Extensive knowledge). (Eriksson and Fries 2020)
Ecosystem services (ESS) attitudes Ecological social (6, *α* = 0.91) *M* = 11.47, SD = 5.42 Production economic (6, *α* = 0.91) *M* = 11.85, SD = 5.58	1) How important is it that your forest contributes to the following? Five-point scale (1 = Not at all important, 5 = Very important) 2) To what extent do you believe that improved birch contributes to the following? Five-point scale (1 = Not at all, 5 = To a great extent) and a possibility to answer Don’t know. Scale: 1–25. (Formative indicators of attitudes, the expectancy value model, Ajzen, 2020) *Ecological social:* Diversity of species (e.g., plants, insects and birds), Preservation of red listed species, Beautiful nature, Recreational forest, Possibilities to pick berries and mushroom for all, Hunting possibilities for you or others following an agreement *Production economic*: Production of timber, Production of pulp, Refined wood products such as veneer and plywood, Biomass for energy (e.g., for production of heat, electricity, fuel), Biomass for production of green carbon molecules (are included in renewable material and chemicals, e.g., textiles, bio char), Economic yield for you
Emotions Negative (3, *α* = 0.91) *M* = 2.04, SD = 1.27 Positive (3, *α* = 0.91) *M* = 3.04, SD = 1.52	To what extent does improved birch elicit the following emotions in you? *Negative:* Fear, Worry, Despair *Positive*: Interest, Joy, Enthusiasm Six-point scale (0 = Not at all, 6 = Very strong) (Eriksson et al. 2020)
Negative beliefs (4, *α* = 0.80) *M* = 2.86, SD = 1.02	To what extent do you believe that improved birch in your forest would imply the following? Great risk for damage by pests and diseases (native, e.g., root rot and invasive pests and diseases), Great risk for damage by browsing, Difficult to manage, Great uncertainty regarding management, economy, etc. Five-point scale (1 = Not at all, 5 = To a great extent) and a possibility to answer Don’t know.
Dependent variables: change intentions	
Tree species composition Broadleaved forest Mixed forest Adopt birch (3, *α* = 0.84)	How likely is it that you would change the way you would manage your forest within a time period of 10 years? *Tree species composition*: Change the composition of tree species *Broadleaved forest*: Increase the share of broadleaved forest through natural regeneration *Mixed forest*: Increased the share of mixed forest through natural regeneration *Adopt birch*: How likely is it that you would plant improved birch on the following land types within a time period of 10 years? Forest land with high productivity Forest land with low productivity In mixed forest with conifers Five-point scale (1 = Not at all likely, 5 = Very likely)

### Analyses

Analyses were conducted in SPSS version 29. To address the first objective, the share of owners with no intention to change (answering 1 on the five-point scale), weak intention (>1 ≤ 3) and strong intention (>3) in relation to the goal intention and the three behavioral intentions were calculated. The McNemar test on paired proportions was used to test whether the intention to adopt improved birch differed significantly from the other behavioral intentions. The owners’ cognitions and emotions associated with improved birch were analyzed using descriptive statistics and t-tests for pairwise comparisons.

The second objective was examined by means of logistic regression analyses with change intentions as dependent variables. The intentions were transformed into binary outcomes (reflecting having an intention (2–5) versus having no intention (1)) since the measure of intention to adopt improved birch was skewed. Place variables (region (dummy, Svealand and Götaland = 1), certification (dummy, certification = 1), membership (dummy, membership = 1), trust, flow of information) and interactions (region*certification, region*membership, certification*membership, certification*trust, certification*flow of information, membership*trust, membership*flow of information, and trust*flow of information) were examined as predictors of change intentions, while controlling for gender, age, forest land, and agricultural land. The interaction terms were created to reflect interactions between the physical and social dimensions, between the social and relational dimensions, between the two social dimensions, and finally between the two relational dimensions. The independent variables were mean centered to avoid problems with collinearity. The models were assessed using the goodness of fit measure of −2 Log likelihood and Pseudo R^2^_N_ and significant interactions were plotted (probabilities). For these plots, three approximately equal size groups were created for flow of information and trust in forest actors, respectively, reflecting low, medium and high levels of each.

The third objective was examined using hierarchical logistic regression analyses. In the first step, risk damage perception, social norm, personal norm, and goal feasibility were included as predictors of change intentions using the same control variables as in the place models. In a second step, goal intention was included together with the general psychological variables as predictors of behavioral intentions, also including control variables. Finally, a separate logistic regression analysis applied to intention to adopt improved birch was used to test the full set of psychological variables, also including specific psychological factors related to improved birch. The independent variables were mean centered. The models were assessed using the goodness of fit measure of −2 Log likelihood and Pseudo R^2^_N_. Bivariate correlations between study variables are displayed in Supplementary Material (Table S2).

## Results

### Change Intentions

Analyses of the first objective showed that the majority of the owners displayed an intention to change tree species composition, 44% a weak intention and 27% a strong intention. Intentions to increase broadleaves through natural regeneration were higher than by adopting improved birch (broadleaves: 43% and 41%, respectively, mixed forest: 44 and 39%, respectively, improved birch: 38% and 6%, respectively) (*z* = −20.74, *p* = 0.001, and −21.54, *p* = 0.001, respectively). The owners displayed little experience of seeing stands of improved birch and little subjective knowledge of the species (Table 1). On average they believed that improved birch contributes slightly more to production/economic ESS than to ecological/social ESS (*t*(1266) = −2.39, *p* = 0.017). Moreover, improved birch evoked more positive than negative emotions among the owners (*t*(1619) = 21.03, *p* = 0.001), and negative beliefs were below the mean of the scale.

### Place and Change Intentions

Results from the logistic regression analyses addressing the second objective are displayed in Table 2. Owning forest in the south region was associated with stronger change intentions. Whereas membership displayed no significant association with either of the intentions, certification was positively associated with intention to change tree species composition and intention to increase broadleaved forest through natural regeneration. In addition, flow of information was positively associated with all intentions, but trust was only associated with intention to adopt improved birch.

**Table 2:** Results from logistic regression analyses of the importance of place dimensions for change intentions

	Tree species composition				Broadleaved forest				Mixed forest				Improved birch			
	*B* (SE)	Wald χ2	*p*	Odds ratio	*B* (SE)	Wald χ2	*p*	Odds ratio	*B* (SE)	Wald χ2	*p*	Odds ratio	*B* (SE)	Wald χ2	*p*	Odds ratio
Constant	1.12 (0.08)	215.03	0.001	3.06	2.13 (0.12)	340.24	0.001	8.38	1.84 (0.10)	360.44	0.001	6.30	−0.32 (0.06)	27.14	0.001	0.73
Region (1 = Svealand, Götaland)	0.66 (0.15)	17.98	0.001	1.92	0.99 (0.17)	33.47	0.001	2.68	0.08 (0.17)	42.56	0.001	2.93	0.35 (0.13)	7.71	0.005	1.42
Membership (1 = Member)	0.13 (0.14)	0.89	0.345	1.14	−0.17 (0.21)	0.63	0.427	0.85	0.06 (0.19)	0.08	0.778	1.06	0.05 (0.11)	0.18	0.674	1.05
Certification (1 = Certification)	0.70 (0.20)	12.70	0.001	2.02	0.77 (0.31)	5.65	0.017	2.09	0.24 (0.25)	0.94	0.332	1.27	0.26 (0.15)	2.86	0.091	1.30
Trust	−0.06 (0.09)	0.46	0.500	0.94	−0.07 (0.13)	0.32	0.570	0.93	0.11 (0.11)	1.01	0.316	1.12	0.21 (0.07)	8.58	0.003	1.23
Flow of information	0.92 (0.12)	60.59	0.001	2.51	1.01 (0.16)	40.13	0.001	2.75	1.23 (0.16)	60.53	0.001	3.44	0.70 (0.09)	61.80	0.001	2.01
Region* Membership	−1.07 (0.27)	15.37	0.001	0.34	−1.19 (0.33)	13.15	0.001	0.30	−0.84 (0.32)	6.75	0.009	0.43	−0.35 (0.26)	1.75	0.185	0.71
Region* Certification	−0.25 (0.44)	0.32	0.572	0.78	1.38 (0.47)	8.54	0.003	3.97	2.12 (0.47)	20.49	0.001	8.36	0.36 (0.34)	1.16	0.282	1.43
Membership* Certification	0.74 (0.36)	4.22	0.040	2.09	−0.51 (0.60)	0.73	0.395	0.60	0.57 (0.51)	1.27	0.259	1.78	0.71 (0.28)	6.55	0.010	2.02
Membership* Trust	−0.19 (0.16)	1.46	0.227	0.83	−0.31 (0.61)	0.26	0.610	0.73	−0.29 (0.19)	2.35	0.126	0.75	−0.11 (0.15)	0.60	0.440	0.89
Membership* Flow of information	−0.10 (0.23)	0.18	0.670	0.91	0.31 (0.34)	0.82	0.365	1.36	−0.80 (0.28)	8.05	0.005	0.41	−0.17 (0.18)	0.87	0.351	0.85
Certification* Trust	−0.20 (0.22)	0.78	0.377	0.82	0.01 (0.33)	0.00	0.973	1.01	−0.45 (0.28)	2.66	0.103	0.64	−0.51 (0.18)	8.48	0.004	0.60
Certification* Flow of information	−0.31 (0.29)	1.11	0.292	0.74	−0.93 (0.42)	5.02	0.025	0.39	0.64 (0.42)	2.30	0.130	1.90	−0.29 (0.20)	2.06	0.152	0.75
Trust*Flow of information	−0.10 (0.14)	0.48	0.489	0.91	−0.27 (0.20)	1.90	0.168	0.76	0.43 (0.16)	7.84	0.005	1.54	0.12 (0.11)	1.39	0.238	1.13
Forest land	0.00 (0.00)	1.27	0.260	1.00	0.00 (0.00)	4.90	0.027	1.00	0.00 (0.00)	1.97	0.161	1.00	0.00 (0.00)	0.05	0.828	1.00
Agricultural land	0.02 (0.01)	5.97	0.015	1.02	0.01 (0.01)	2.43	0.119	1.01	0.01 (0.01)	2.77	0.096	1.01	0.01 (0.00)	2.30	0.130	1.01
Gender (1 = Women)	−0.23 (0.12)	3.57	0.059	0.79	−0.16 (0.15)	1.14	0.286	0.85	−0.38 (0.14)	6.79	0.009	0.69	−0.38 (0.11)	11.58	0.001	0.69
Age	−0.03 (0.00)	39.01	0.001	0.97	−0.01 (0.01)	3.69	0.055	0.99	0.00 (0.01)	0.11	0.739	1.00	−0.02 (0.00)	17.72	0.001	0.98
−2 Log likelihood	1657.78				1212.41				1286.00				2064.18			
Pseudo R^2^_N_	0.24				0.21				0.22				0.15			

**Fig. 2:**
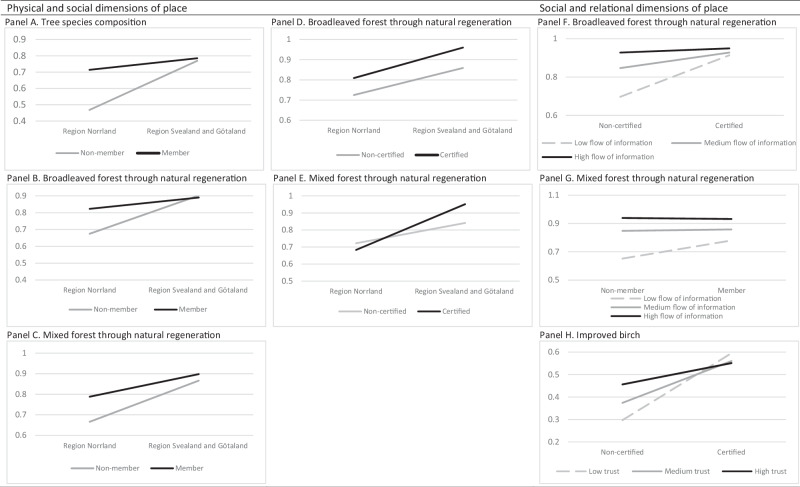
Change intentions as a function of physical and social dimensions of place (**A**–**E**), and social and relational dimensions of place (**F**–**H**) (Probabilities). **A**–**C** Interactions between Region and Membership in forest owner association. **D**, **E** Interactions between Region and Certification. **F** Interaction between Certification and Flow of information, **G** Interaction between Membership in forest owner association and Flow of information. **H** Interaction between Certification and Trust in forest actor

Significant interactions were found between the physical and social place dimensions, between the social and relational place dimensions (Fig. 2), and among the different social as well as relational place dimensions (Supplementary Material, Fig. S1). Results revealed that members in a forest owner organization displayed a stronger intention to change tree species composition, increase broadleaved forest, and mixed forest in the north region, but membership displayed no association with these intentions in the south region (Fig. 2). Moreover, certification was more strongly associated with intention in the models of broadleaved and mixed forest in the south compared to the north region, particularly evident in the mixed forest model. The relationship between relational variables and intentions was furthermore dependent on the social place dimensions in three of the models, though in slightly different ways. For example, a high flow of information was associated with a stronger intention to increase broadleaved forest among non-certified owners compared to certified owners, and a higher trust in forest actor was associated with a stronger intention to adopt improved birch among non-certified owners, while trust did not matter among certified owners. Certification and membership (i.e., the social place dimensions) interacted in the models of tree species composition and improved birch (Fig. S1). For example, the intention to adopt improved birch was stronger among certified owners, and this effect was more pronounced among members. Trust and flow of information also interacted in relation to intention to increase mixed forest with the strongest intention found among owners with both a higher trust and a higher flow of information. The Pseudo R^2^_N_ was above 0.20 for intentions to change tree species composition, increase broadleaved forest and mixed forest through natural regeneration, but lower (0.15) for intention to adopt improved birch.

### Psychological Factors and Change Intentions

In analyses of the third objective, the first step of the hierarchical logistic regression analyses revealed that damage risk perceptions, personal norm and goal feasibility were significantly associated with all change intentions (Table 3). Social norm was significantly associated with intention to change tree species composition and adopt improved birch. The general psychological factors were most important for the intention to change tree species composition (Pseudo R^2^_N_ = 0.31) but less important for the behavioral intentions (Pseudo R^2^_N_ = 0.20, 0.19, 0.16, respectively). In the second step, adding goal intention to the predictors of behavioral intentions revealed that only goal feasibility together with intention to change tree species composition were significant predictors of intention to increase the share of broadleaved and mixed forest. In the model of improved birch, damage risk perception, social norm, and goal feasibility remained significant in addition to the goal intention. The goodness of fit was improved for the models of broadleaved and mixed forest (Pseudo R^2^_N_ = 0.43, 0.42, respectively), supporting the importance of goal intention for these behavioral intentions, and to a lesser extent for intention to adopt improved birch (Pseudo R^2^_N_ = 0.23).

**Table 3:** Results from hierarchical logistic regression analyses of the importance of general psychological factors for change intentions

	Tree species composition				Broadleaved forest				Mixed forest				Improved birch			
	*B* (SE)	Wald χ2	*p*	Odds ratio	*B* (SE)	Wald χ2	*p*	Odds ratio	*B* (SE)	Wald χ2	*p*	Odds ratio	*B* (SE)	Wald χ2	*p*	Odds ratio
Constant	1.22 (0.07)	282.66	0.001	3.37	2.07 (0.09)	511.16	0.001	7.94	1.92 (0.09)	494.13	0.001	6.81	−0.28 (0.06)	26.85	0.001	0.75
Damage risk perception	0.77 (0.08)	84.86	0.001	2.17	0.38 (0.09)	17.00	0.001	1.47	0.26 (0.09)	9.09	0.003	1.30	0.37 (0.07)	30.99	0.001	1.45
Social norms	0.22 (0.08)	8.62	0.003	1.25	0.03 (0.09)	0.09	0.765	1.03	0.05 (0.09)	0.32	0.574	1.05	0.19 (0.06)	8.99	0.003	1.21
Personal norms	0.47 (0.07	40.49	0.001	1.60	0.43 (0.09)	20.88	0.001	1.54	0.33 (0.09)	14.44	0.001	1.39	0.18 (0.06)	10.80	0.001	1.18
Goal feasibility	0.42 (0.07)	38.76	0.001	1.53	0.57 (0.08)	51.44	0.001	1.77	0.60 (0.08)	62.01	0.001	1.83	0.29 (0.08)	13.07	0.001	1.47
Forest land	0.00 (0.00)	1.48	0.224	1.00	0.00 (0.00)	0.13	0.714	1.00	0.00 (0.00)	2.05	0.153	1.00	0.00 (0.00)	1.30	0.254	1.00
Agricultural land	0.02 (0.01)	10.27	0.001	1.03	0.02 (0.01)	4.17	0.041	1.02	0.02 (0.01)	5.43	0.020	1.02	0.01 (0.00)	3.88	0.049	1.01
Gender	−0.62 (0.13)	21.71	0.001	0.54	−0.37 (0.16)	5.56	0.018	0.69	−0.56 (0.15)	14.50	0.001	0.57	−0.63 (0.11)	30.82	0.001	0.53
Age	−0.02 (0.00)	24.93	0.001	0.98	0.00 (0.01)	0.20	0.652	1.00	0.01 (0.01)	5.20	0.023	1.01	−0.01 (0.00)	5.85	0.016	0.99
−2 Log likelihood	1518.35				1180.89				1268.34				1975.55			
Pseudo R^2^_N_	0.31				0.20				0.19				0.16			
Constant					2.56 (0.13)	403.86	0.001	12.99	2.33 (0.11)	425.07	0.001	10.32	−0.36 (0.06)	36.71	0.001	0.70
Damage risk perception					0.02 (0.11)	0.03	0.874	1.02	−0.14 (0.10)	1.75	0.185	0.87	0.21 (0.07)	9.12	0.003	1.24
Social norms					−0.10 (0.10)	0.92	0.339	0.91	−0.05 (0.10)	0.31	0.580	0.95	0.15 (0.07)	4.87	0.027	1.16
Personal norms					0.17 (0.10)	2.91	0.088	1.19	0.07 (0.09)	0.50	0.480	1.07	0.09 (0.06)	2.46	0.117	1.09
Goal feasibility					0.40 (0.09)	21.57	0.001	1.50	0.46 (0.08)	30.26	0.001	1.58	0.31 (0.07)	22.27	0.001	1.36
Goal intention: Tree species composition					2.99 (0.22)	192.55	0.001	19.85	2.91 (0.20)	210.39	0.001	18.34	1.45 (0.15)	88.93	0.001	2.26
Forest land					0.00 (0.00)	2.47	0.116	1.00	0.00 (0.00)	0.56	0.455	1.00	0.00 (0.00)	0.59	0.443	1.00
Agricultural land					0.01 (0.01)	0.49	0.482	1.01	0.01 (0.01)	1.00	0.319	1.01	0.00 (0.00)	0.37	0.242	1.00
Gender (1 = Women)					−0.03 (0.18)	0.02	0.883	0.98	−0.27 (0.17)	2.51	0.113	0.77	−0.54 (0.12)	20.89	0.001	0.58
Age					0.01 (0.01)	4.68	0.030	1.01	0.03 (0.01)	24.85	0.001	1.03	−0.01 (0.01)	1.22	0.269	1.00
−2 Log likelihood					910.32				985.46				1846.50			
Pseudo R^2^_N_					0.43				0.42				0.23			

Only damage risk perception and goal intention remained significant after adding the specific psychological factors as predictors of intention to adopt improved birch (Table 4). In addition, more experience and subjective knowledge of improved birch, considering improved birch to have positive impacts on ecological social ESS, as well as experiencing positive emotions in response to improved birch, and weaker negative beliefs were associated with a stronger intention to adopt improved birch. With the specific psychological factors, the goodness of fit improved considerably (Pseudo R^2^_N_ = 0.38).

**Table 4:** Results from a logistic regression analysis of general and specific psychological factors for intention to adopt improved birch

	Improved birch			
	*B* (SE)	Wald χ2	*p*	Odds ratio
Constant	−0.10(0.09)	1.26	0.262	0.91
Damage risk perception	0.24(0.10)	5.24	0.022	1.27
Social norms	0.11(0.09)	1.29	0.256	1.11
Personal norms	0.08(0.09)	0.84	0.359	1.08
Goal feasibility	0.13(0.09)	2.00	0.157	1.14
Goal intention: tree species composition	0.87(0.21)	17.26	0.001	2.40
Experience	0.25(0.10)	6.73	0.009	1.28
Subjective knowledge	0.26(0.10)	7.53	0.006	1.30
Ecological social attitude	0.04(0.02)	5.05	0.025	1.04
Production economic attitude	0.01(0.02)	0.57	0.449	1.01
Positive emotions	0.52(0.07)	55.48	0.001	1.68
Negative emotions	−0.12(0.07)	3.29	0.070	0.89
Negative beliefs	−0.18(0.09)	4.07	0.044	0.84
Forest land	0.00(0.00)	1.65	0.056	1.00
Agricultural land	0.00(0.00)	0.02	0.895	1.00
Gender (1 = Women)	−0.12(0.17)	0.46	0.498	0.89
Age	−0.01(0.01)	2.32	0.128	0.99
−2 Log likelihood	1007.60			
Pseudo R^2^_N_	0.38			

## Discussion

This study applied a novel interdisciplinary approach to study dynamics in the decision context and decision-making associated with a transition towards diversified management among forest owners in Sweden. Results revealed that the intention to adopt improved birch was weaker than the intention to increase broadleaved and mixed forest through natural regeneration, indicating that management aligned with current practices is more likely to be adopted. Increasing broadleaves through natural regeneration may not only result from a conscious decision to change tree species composition, but some other goal intention, since fewer owners displayed such goal intention compared to behavioral intentions to increase broadleaves through natural regeneration. Fast-growing forest is not generally considered important to Swedish forest owners (Bergkvist et al. 2024), yet improved birch constitutes a new opportunity. Whereas increasing the share of improved birch aligns with the production focus of the forest sector in Sweden (Andersson and Keskitalo 2018) and changes that supports the logics of involved actors are more likely to occur (Banos et al. 2022), regeneration of improved birch is still a major shift from current management practices of broadleaves since it requires improved plant material, planting etc. and therefore increased costs for regeneration. As has been noted in relation to CCF (Mason et al. 2022), lack of awareness and management knowledge among owners may, at least initially, prevent the expansion of novel options at the initiation of a transition.

The importance of different place dimensions and interactions for intentions to change management was confirmed, thereby supporting that dynamics in the decision-making context of owners play a role for management change. For example, the south region and a higher flow of information from different sources (e.g., other owners, advisors, and the SFA) were associated with stronger intentions to change, supporting the importance of both the physical and relational dimensions of place as relevant for change in management. Importantly, however, interactions were also significant. Membership in a forest owner association and certification reflecting social dimensions of place displayed different associations with intentions depending on region. In the north region, membership in a forest owner association was associated with stronger change intentions (except in relation to improved birch) but the same pattern was not found in the south region. In contrast, certification was associated with a stronger intention to increase mixed forests in the south but not in the north region. Hence, membership and certification may not reflect the same social conditions for owners in different regions, and this may be due to differences between organizations in the different regions (Kronholm 2015) or derive from the social—physical nexus (e.g., certification standards may have different implications depending on climatic conditions). South region and flow of information was also associated with a stronger intention to adopt improved birch, but so was a higher trust in forest actors, and the interactions differed from the other models. For example, the interaction between trust and certification indicates that trust plays a greater role for intention to adopt improved birch among non-certified compared to certified owners. This result suggests that during the consideration of novel management options, trust—reflecting a willingness to rely on others (Rousseau et al., (1998))—is important, and particularly so outside the social context created by certification schemes. Moreover, being certified and a member of a forest owner association was associated with a stronger intention to adopt improved birch. Such an additive pattern was not found in relation to the intention to increase broadleaved and mixed forests through natural regeneration in this study but has been shown in relation to management in previous research (Bergkvist et al. 2024). Several measures in this study did not distinguish between scales (e.g., trust in owners and the SFA) but displayed high internal reliability, yet future studies may want to consider both place dimensions and scales (e.g., local and national) to better understand the owners’ decision-making context.

Both seeing a need for change (a higher damage risk perception) and facilitating variables (a stronger personal norm, goal feasibility, as well as experience, cognitions, and emotions linked to a specific behavioral option) were found to be associated with stronger change intentions. This supports a need to understand the decision-making process preceding management change. The general psychological variables were more strongly associated with the goal intention than with the behavioral intentions. In addition, the reduced impact of general psychological variables for the intention to adopt improved birch when the specific psychological variables were added confirms the importance of specific psychological variables for understanding why a certain option is selected over another. Norms have been suggested to play a role for the spread of a transition into different geographies (Köhler et al. 2019), and when forestry is deregulated, as in the case of Sweden, norms should be important for management (Andersson and Keskitalo 2018). However, the role of norms for management has not been extensively studied (Floress et al. 2019). Our study provides insights on how normative influences are relevant for management change. A stronger social norm, signaling that increasing broadleaves is accepted, was associated with the intention to change tree species composition and the intention to adopt improved birch. In addition, personal norm, reflecting perceptions that increasing broadleaves is the right thing to do, was found to be important for all behavioral intentions, yet less relevant when the goal intention had been added to the models. More experience and subjective knowledge of improved birch, a stronger belief in the ecological and social benefits of improved birch (but not production economic benefits), stronger positive emotions, and weaker negative beliefs were all associated with the intention to adopt improved birch. Hence, management decisions are not only a result of cognitively based factors (e.g., forest values, beliefs and knowledge) as shown in previous studies (Eriksson and Fries 2020), but also emotions. The importance of positive (but not negative) emotions is a novel finding in studies of forest management.

There are limitations associated with this study worth considering. The data is based on a representative sample of forest owners in Sweden, but there are deviations from the population and even with statistical weights to control for deviations in gender, age and forest size, the descriptive results in particular, should be interpreted with caution (the population values may be slightly higher or lower). The overall pattern concerning how the predictors are associated with dependent variables can be considered less vulnerable to sample deviations, given the theoretically based analyses, large sample, and reliable indicators. Even though the theoretical framework emphasizes that change is a process in a dynamic setting, the data is cross-sectional and analyses correlational. Greater theoretical integration is also needed, for example, connecting different place dimensions with psychological processes at different stages of behavioral change. Future studies should consider collecting longitudinal data to enable direct examination of change processes over time, and experimental studies are needed to clarify causal mechanisms involved in transitions. By combining research of forest owners with in-depth understanding of the study context, hypotheses can be outlined and tested, thereby developing a place-based understanding of management decisions. While the focus of this study is appropriate during the initial phase of a transition, further on, also the later stages of behavioral change need to be examined for an understanding of when intention translates into behaviors.

There is an increased interest in understanding owners’ management decisions (Epanshin-Niell et al. 2022). Despite that both conditions and the decision-making process are key to understanding these decisions, integrations of geographical and psychological perspectives are rare. Policy is a key dimension of transition dynamics (Geels 2010), and research such as this study may help to guide the development of policies encouraging management transitions, in this case, a transition from a conifer dominated forest to a forest with more broadleaves. Given that membership in owner organizations and certification schemes play a role for change intentions, these organizations are likely important channels to reach forest owners in different contexts, but since a high information flow to the owners, and in some cases trust in forest actors, may be equally important, a diverse set of channels (e.g., forest networks) and direct outreach is needed to connect with more owners. Management options that are novel and either not aligning with norms or costly (monetarily or otherwise) are more likely to require strong government measures to initiate or encourage change (Eriksson 2018b). In New-Zeeland, monetary incentives, but also non-monetary incentives such as the provision of seedlings have been found to be key for native afforestation (Polyakov et al. 2024). A policy mix for a transition towards more broadleaves needs to be based on owner perspectives, but also other stakeholders, since changes in landcover may be resisted by some stakeholders (Dhubháin et al., (2009)). In addition, systemic factors such as current policy, government capacities, the forest sector, and the market need to be considered.

## Supplementary information

Supplementary materials

## Data Availability

The datasets generated and analysed during the current study are available from the corresponding author on reasonable request.
