# Supplementary material for: Insights on Place and Psychological Dynamics for Private Forest Owners’ Management Transitions: A Study on Increasing the Share of Broadleaves in Sweden
Source: Environ Manage. 2025 Aug 8;75(10):2841–54. doi: 10.1007/s00267-025-02234-x (PMC12457465; doi:10.1007/s00267-025-02234-x)
Supplement: Supplementary file 1 — Supplementary materials [file 267_2025_2234_MOESM1_ESM.docx]

**Supplementary materials**

Table S1. Descriptives for the sample of private forest owners weighted on gender, age and size of forest holding.

| Gender (women) | 39% |
| --- | --- |
| Mean age | 64 years (SD = 14.5) |
| Education | 47% university degree |
| Forest estate region | N. Norrland: 20%  S. Norrland: 13%  Svealand: 28%  Götaland: 38% |
| Place (rural) | 69% |
| Owner category (resident) | 40% |
| Size of forest holding | 71.5 ha (SD = 238.5)  5-20 ha: 40%, 21-50 ha: 31%, 51-200 ha:23%, >200: 5% |
| Size of agricultural land | 6.8 ha (SD = 18.5) |
| Member in forest owner association | 47% |
| Certified (FSC, PEFC or both) | 29% |

Table S2. Bivariate correlations among study variables.

|  | Reg | Memb | Cert | Trust | Info | Risk | SN | PN | GoalF | GoalI | Exp | Know | EcoSoc A | ProdEcon A | Pos E | Neg E | Neg B | Broad | Mix |
| --- | --- | --- | --- | --- | --- | --- | --- | --- | --- | --- | --- | --- | --- | --- | --- | --- | --- | --- | --- |
| Memb | .08*** |  |  |  |  |  |  |  |  |  |  |  |  |  |  |  |  |  |  |
| Cert | .24*** | .31*** |  |  |  |  |  |  |  |  |  |  |  |  |  |  |  |  |  |
| Trust | .05* | .07** | .12*** |  |  |  |  |  |  |  |  |  |  |  |  |  |  |  |  |
| Info | .11*** | .17*** | .27*** | .11*** |  |  |  |  |  |  |  |  |  |  |  |  |  |  |  |
| Risk | .20*** | .15*** | .26*** | .11*** | .16*** |  |  |  |  |  |  |  |  |  |  |  |  |  |  |
| SN | .17*** | .10*** | .09*** | .16*** | .07** | .14*** |  |  |  |  |  |  |  |  |  |  |  |  |  |
| PN | .12*** | .03 | .08*** | .08*** | .11** | .13*** | .34*** |  |  |  |  |  |  |  |  |  |  |  |  |
| GoalF | .14*** | .08** | .19*** | .15*** | .16*** | .17*** | .26*** | .39*** |  |  |  |  |  |  |  |  |  |  |  |
| GoalI | .21*** | .11*** | .24*** | .07** | .26*** | .27*** | .21*** | .28*** | .32*** |  |  |  |  |  |  |  |  |  |  |
| Exp | .13*** | .07** | .13*** | .10*** | .17*** | .12*** | .19*** | .13*** | .14*** | .18*** |  |  |  |  |  |  |  |  |  |
| Know | .12*** | .11*** | .23*** | .10*** | .34*** | .15*** | .13*** | .07** | .17*** | .19*** | .42*** |  |  |  |  |  |  |  |  |
| EcoSoc A | .08** | .05 | .00 | .15*** | .04 | .15*** | .17*** | .25*** | .10*** | .08** | .12*** | .08** |  |  |  |  |  |  |  |
| ProdEcon A | .09*** | .14*** | .24*** | .25*** | .26*** | .27*** | .17*** | .08** | .21*** | .22*** | .18*** | .28*** | .39*** |  |  |  |  |  |  |
| Pos E | .11*** | .06** | .18*** | .18*** | .25*** | .19*** | .24*** | .21*** | .24*** | .32*** | .22*** | .37*** | .37*** | .46*** |  |  |  |  |  |
| Neg E | .00 | -.08** | -.04 | -.05 | -.07** | .00 | .07** | .17*** | .01 | .11*** | -.02 | -.08** | -.11*** | -.17*** | .09*** |  |  |  |  |
| Neg B | .04 | .04 | .08* | .07* | .02 | .24*** | .00 | .05 | .05 | .08** | -.03 | -.07* | .02 | .09** | -.02 | .24*** |  |  |  |
| Broad | .20*** | .06* | .17*** | .07** | .23*** | .15*** | .13*** | .22*** | .30*** | .54*** | .13*** | .10*** | .14*** | .14*** | .24*** | .04* | .02 |  |  |
| Mix | .20*** | .08*** | .14*** | .06** | .24*** | .13*** | .12*** | .20*** | .30*** | .53*** | .08*** | .10*** | .15*** | .14*** | .26*** | .08*** | .05 | .77*** |  |
| I birch | .11*** | .06** | .16*** | .12*** | .25*** | .18*** | .17*** | .18*** | .24*** | .35*** | .25*** | .33*** | .19*** | .30*** | .47*** | -.09*** | -.05 | .23*** | .24*** |

Calculation method: Phi for two dummy variables. Point biserial correlation for one dummy and one continuous variable. Pearson correlation r, for two continuous variables. Reg = Region Dummy, Memb = Membership Dummy, Cert = Certification Dummy, Trust = Trust Dummy, Info = Flow of information, Risk = Damage risk perception, SN = social norms, PN = personal norms, Goal F = Goal feasibility, Goal I = Goal intention Dummy, Exp = Experience , Know = Subjective knowledge, EcoSoc A = Ecological social attitude, ProdEcon A = Production economic attitude, Pos E = Positive Emotions, Neg E = Negative emotions, Neg B = Negative beliefs, Broad = Intention to increase the share of broadleaves Dummy, Mix = Intention to increase the share of mixed forest Dummy, I birch = Intention to adopt improved birch Dummy.

| Different social dimensions of place | Different relational dimensions of place |
| --- | --- |
| Panel A. Tree species composition | Panel C. Mixed forest through natural regeneration |
| Panel B. Improved birch |  |

Figure S1. Change intentions as a function of different social dimensions of place (panel A-B) and different relational dimensions of place (panel C) (Probabilities). Panel A-B) Interactions between Membership in forest owner association and Certification. Panel C) Interaction between Trust in forest actors and Flow of information.
